# Development of a Multiuser Interactive Health Response Application (MITHRA) for depression in women from a community-based organisation in India

**DOI:** 10.1192/bjo.2025.8

**Published:** 2025-03-25

**Authors:** Johnson-Pradeep Ruben, Dhinagaran Devadass, B. Ramakrishna Goud, Yesenia Navarro-Aguirre, Bharat Kalidindi, Abijeet Waghmare, Tony Raj, Krishnamachari Srinivasan, Pamela Y. Collins, Amritha Bhat

**Affiliations:** 1 St John’s Medical College, Bengaluru, India; 2 St John’s Research Institute, Bengaluru, India; 3 Department of Anthropology, University of Washington, Seattle, Washington, USA; 4 Department of Global Health, University of Washington, Seattle, Washington, USA; 5 Department of Psychiatry and Behavioral Sciences, University of Washington, Seattle, Washington, USA

**Keywords:** Depression, mental health apps, self-help groups, rural area, women

## Abstract

**Background:**

In India, women in rural areas have high rates of depression. They have poor access to mental healthcare resources and, hence, mental health symptoms remain largely unaddressed. Existing mobile telephone applications (apps) do not engage end-users, lack local language options, may not be socioculturally relevant and do not use audiovisual formats. We thus developed a mobile mental health app, Multiuser Interactive Health Response Application (MITHRA), to screen and provide brief behavioural intervention for mild to moderate depression among rural women attending self-help groups (SHGs) in India.

**Aims:**

This qualitative study explores the process and findings of focus groups conducted with SHG administrators and women to inform the iterative development of the MITHRA app.

**Method:**

In total, 22 participants were interviewed (17 SHG participants and five administrators), and a thematic analysis of the data was conducted using the acceptability of interventions framework.

**Results:**

Frequent themes across the focus groups were affective attitude, burden, self-efficacy and perceived effectiveness. All women showed a positive attitude towards the app and depression interventions, while older women demonstrated less self-efficacy in using mobile mental health apps.

**Conclusions:**

MITHRA is a promising app in the management of mild to moderate depression in women in SHG. With adequate training and education of family members, MITHRA has the potential to identify and treat women with mild to moderate depression.

Mental health is an important determinant of quality of life; however, it is often neglected, especially in women. One in ten women in rural India experiences common mental disorders such as anxiety and depression and, due to poor access to mental healthcare resources, these mental health symptoms remain largely unaddressed.^1^ Screening and treatment for depression through brief interventions using available resources is one way to address this treatment gap.^2^ Mobile applications (apps) could play an important role: mobile app-based electronic decision support systems for healthcare workers are associated with improved mental health outcomes, including increased access to mental health services, reduced stigma and improved quality of life among rural Indian women.^3^ However, most apps available for patient use in India are focused on conditions such as diabetes or hypertension, with no culturally appropriate apps for mental health.^4^ Mental health apps have mainly been focused on physicians and community health workers^4^ to date, and not on women with depression or anxiety.

## Multiuser Interactive Health Response Application (MITHRA)

**Fig. 1:**
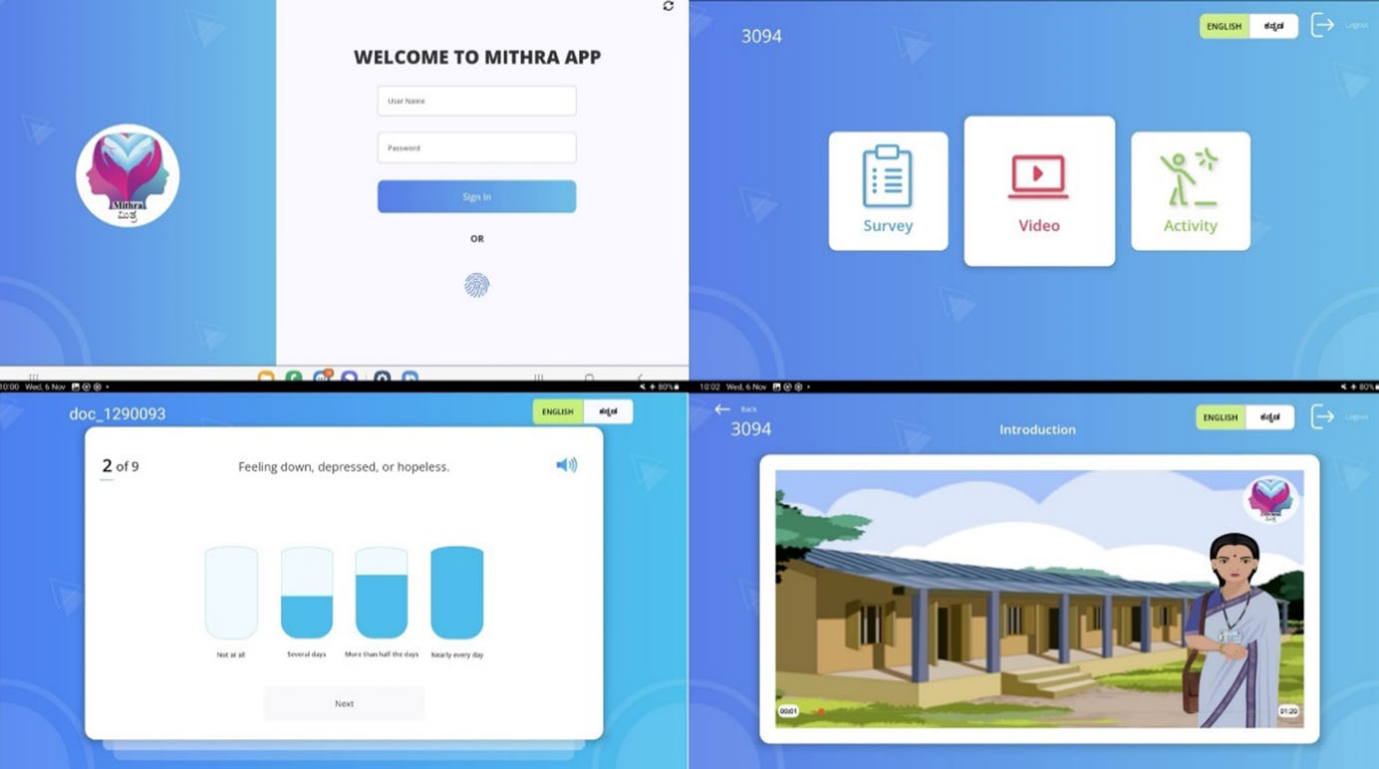
Multiuser Interactive Health Response Application (MITHRA) screenshots.

In response to this need, we developed a mobile mental health app, MITHRA, to screen and provide brief behavioural intervention for depression among rural women attending self-help groups (SHGs) in India.^5^ Women in rural India have limited access to smartphones due to shared ownership of phones,^5^ and the app was intended to be used on a tablet shared among women attending SHGs. This app was iteratively developed based on feedback collected from focus group discussions with SHG members and administrators. These focus groups helped us obtain end-users’ input in app development, including input on app structure, content, design and quality. The mobile app termed MITHRA (see screenshots in Fig. 1), which means ‘friend’ in the regional language Kannada, will support the identification, initial treatment and referral of women with depression and is designed to address barriers such as illiteracy and lack of access to a personal mobile device. Women will complete depression screening using patient health questionnaire PHQ-9.^6^ Based on the severity of scores, the app will deliver different modules, including education about depression and activity scheduling, based on the healthy activity programme (HAP), a short, evidence-based psychological intervention that is acceptable, efficacious and cost-effective in the treatment of depression and has been tested in rural India.^7,8^ Modules will include instructions on managing stress, breathing techniques, improving sleep, getting active and problem-solving. This ensures that most women with mild to moderate depression receive initial treatment without having to overcome transportation barriers. Women with severe depression will receive instructions on how to contact the primary health centre for treatment. If suicidal ideation is endorsed (question 9 on PHQ-9), the community health worker will receive an alert to assess for imminence and to ensure the appropriate level of follow-up. This paper describes the process and findings of the focus groups conducted to inform the iterative development of the MITHRA app. We aimed to conduct focus group discussions (FGDs) to obtain the perspectives of SHG participants and administrators on the use of an app in identifying and treating depression among SHG participants.

## Method

### Study site

The study was conducted in Anekal taluk (subdistrict administrative block of a state in India), Karnataka, which is approximately 25 miles from Bengaluru. It has a population of 517,575, with 48% being female. Among the 40 villages in Anekal taluk, with a total population of 9724, ten have functional women’s SHGs (https://villageinfo.in/karnataka/bangalore/anekal/m-medihalli.html). SHGs are run by the Centre for Integral Rural Welfare (CIRW), a non-governmental organisation that works with marginalised women to empower them. The primary aim of CIRW is to help people become agents of social change. As a part of this mission, it supports schools and provides training to women through tailoring institutes. In the community, CIRW manages more than 40 women’s SHGs to facilitate microfinancing and social empowerment.

### Research team

The FGDs were facilitated by J.-P.R. and B.R.G., male members of the research team. J.-P.R. (MD in Psychiatry) is a consultant psychiatrist, B.R.G. (MD in Community Medicine) is a public health consultant, D.D. (BDS and MBA-HCS) is the information technology consultant, B.K. (BAMS and MPH) is a research coordinator for this study and A.W. (MBBS, Adv. Dip. in Bioinformatics, MBA) is an information technology consultant. All researchers were trained in qualitative research methodology. The FGD facilitators and first authors are aware of their positionality as clinicians who have been working to deliver healthcare to women in rural India. FGD participants of the SHG did not know any of the researchers; they were briefed by their leaders about the purpose of the FGD. FGD participants, comprising administrators, knew the researchers well.

### Theoretical framework

We used the theoretical framework of acceptability,^9^ which includes seven component constructs: affective attitude, burden, ethicality, intervention coherence, opportunity costs, perceived effectiveness and self-efficacy. These constructs were used to develop themes for the thematic analysis. Affective attitude is defined as ‘how an individual feels about taking part in an intervention’. Burden is the perceived amount of effort required to participate in the intervention. Ethicality refers to how well the intervention fits within the respondents’ value system. Intervention coherence refers to the extent to which the participant understands the intervention and how it works. Opportunity cost is the extent to which benefits, profits or values must be given up to engage in the intervention. Perceived effectiveness is the extent to which the intervention is perceived as likely to achieve its purpose. Self-efficacy refers to the participant’s confidence that they can perform the behaviour(s) required to participate in the intervention.

### Participants

The authors assert that all procedures contributing to this work comply with the ethical standards of the relevant national and institutional committees on human experimentation, and with the Helsinki Declaration of 1975 as revised in 2008. All procedures involving human subjects/patients were approved by the University of Washington and St. Johns’ Research Institute, in June and August 2020 respectively.

We used purposive sampling to recruit female SHG participants, residents of the village and community health workers and administrators of the SHGs who had been in their current role for at least 6 months. Women diagnosed with a severe mental illness such as bipolar disorder or schizophrenia, and those with a history of suicide attempt or severe alcohol or substance use in the previous 6 months, were excluded because the intended use of the app is for treatment of mild to moderate depressive symptoms. Those who were unable to participate in the informed consent discussion were also excluded. Written informed consent was obtained face to face from all participants.

Participants were recruited via (a) advertisements, (b) flyers, (c) word of mouth and (d) community health workers (CHWs). CHWs were recruited from SHGs by contacting them through the parent CIRW. Across two focus groups for SHG women there were 17 participants, and in one focus group for administrators there were five participants (two males and three females). Based on previous research, this sample size is expected to support reaching saturation of data collected.^10^ Participants for the administrator group were recruited on 30 November 2021, and SHG participants on 21 December 2021.

### Data collection

We developed an interview guide (see Supplementary Material available at https://doi.org/10.1192/bjo.2025.8) with questions and probes based on the theoretical framework of acceptability. We conducted three FGDs, two with SHG participants and one with SHG community health workers and administrators, each lasting 60 min. In FGDs with SHG participants, we obtained inputs on the design and features of the proposed app to be installed on a tablet, including specifications such as audio enablement, simplified touchscreen and a follow-up decision algorithm. We also included questions about screening for depression using PHQ-9, length of modules to be viewed and women’s preferences regarding viewing HAP modules at home or at the SHG. All FGDs were conducted in the office space of Jnana Jyothi (CIRW) on the same day, but in three different private rooms. FGDs were audio recorded, transcribed, translated into English and cross-verified by the researchers who conducted the FGD. Field notes were made and reviewed during coding and analysis.

### Data analysis

Each transcript was reviewed by two coders. We used Dedoose version 9.2.22 (a cloud application for managing, analysing, and presenting qualitative and mixed method research data: SocioCultural Research Consultants, LLC, Los Angeles, CA, USA; www.dedoose.com) for analysis, and a thematic analysis approach using constructs from the theoretical framework of acceptability to code transcripts, generating new codes as needed. Discrepancies in coding were discussed and, based on the consensus of all reviewers, themes were merged or a new theme was generated. We used the Consolidated criteria for reporting qualitative research checklist for the reporting of qualitative research.

### Trial registry

The study is registered in the Clinical Trial Registry of India (no. CTRI/2020/12/029844, registered 16 December 2020) and at clinicaltrials.gov (no. NCT04480021).

## Results

### Description of study sample

More than 50% of SHG participants in the study were aged 30–50 years. The majority were uneducated (49%), were involved in unskilled labour and worked for daily wages (55%); more than half had a household income of less than Rs. 15 000 per month. Notably, the age range of participants varied considerably between the two FGDs of SHG members. In group A, most of the participants were younger (42.9% aged 18–26 years), while in Group B, 50% were aged 28–34 years. Participant characteristics are shown in Table 1.

**Table 1:** Demographics of FGD participants (SHG women)

	Group A		Group B		Total	
	*n*	%	*n*	%	*n*	%
Total FGD participants	8	47	9	53	17	100
Age (years)						
18–25	2	25	1	11	3	18
26–30	1	13	2	22	3	18
31–35	2	25	5	56	7	41
36–40	2	25	1	11	3	18
55–60	1	13	0	0	1	6
Education						
No schooling	1	13	0	0	1	6
High school	3	38	4	44	7	41
PUC	3	38	1	11	4	24
Degree	1	13	1	11	2	12
Masters	0	0	1	11	1	6
NA	0	0	2	22	2	12
Family type						
Nuclear	4	50	6	67	10	59
Joint	3	38	3	33	6	35
NA	1	13	0	0	1	6
Marital status						
Married	7	88	9	100	16	94
Unmarried	1	13	0	0	1	6
Number of adult family members						
1–2	4	50	3	33	7	41
3–4	3	38	4	44	7	41
7–8	1	13	0	0	1	6
NA	0	0	2	22	2	12
Number of child family members						
1–2	6	75	7	78	13	76
3–4	1	13	1	11	2	12
5–6	1	13	0	0	1	6
NA	0	0	1	11	1	6
Income (Rs.)						
1000–5000	1	13	2	22	3	18
5001–10 000	4	50	3	33	7	41
10 001–15 000	2	25	3	33	5	29
NA	1	13	1	11	2	12
Average income (Rs.)	10 704		10 000		10 500	
Wage earners						
1–2	5	63	5	56	10	59
3–4	3	38	2	22	5	29
5–6	0	0	1	11	1	6
NA	0	0	1	11	1	6
Occupation						
Housewife	4	50	6	67	10	59
Daily waged labour	0	0	2	22	2	12
Tailor	2	25	0	0	2	12
Asha worker	1	13	1	11	2	12
Anganwadi worker	1	13	0	0	1	6

FGD, focus group discussion; SHG, self-help group; PUC, pre-university course; NA, not applicable; Rs, Rupees.

Five administrators from CIRW were part of the focus group; all were educated and serving various roles in the organisation including coordinator, teacher and director. None had previous training in mental health work. Administrator characteristics are shown in Table 2.

**Table 2:** Demographics of FGD participants (SHG administrators)

	*n*	%
Total participants	5	100
Gender		
Female	3	60
Male	2	40
Education		
High school	3	60
Graduation	1	20
Postgraduation	1	20
Age group (years)		
30–50	3	60
51 and above	2	40
Total	5	100
Role		
Coordinator	3	60
Teacher	1	20
Director	1	20
Previous training in mental health		
Yes	0	0
No	5	100

FGD, focus group discussion; SHG, self-help group.

The results of our analysis of the three focus groups are summarised in Table 3. The most frequent themes across the focus groups were affective attitude (25 codes), burden (21 codes) and self-efficacy (15 codes). Another frequent theme was perceived effectiveness related to app usage (19 codes).

**Table 3:** Themes, subthemes and sample quotes

Themes (definitions)	Subthemes	Sample quotes
**Affective attitude** (how an individual feels about the intervention)	Hesitation to use individual devices in their homes	‘… they may keep it somewhere and forget it, if there are children in the house they may throw it or may break it …’ (Admin.) ‘… and there is a fear of ruining digital objects if they are touched’
	Need to defer to leadership when used in the SHG context	‘The (leader) is the knowledgeable person in the self-help group if you give it to them, everyone will go and take from them’ (Admin.)
**Burden**(the perceived amount of effort that is required to participate in the intervention)	Higher burden among older users or those with lower digital literacy	‘Having audio can help with understanding, but it might still be difficult for old people’ (Admin.)‘… if people already know (use of mobile apps) they can understand … old people find it difficult’
**Ethicality** (the extent to which the intervention is a good fit with an individual’s value system)	Questions about depressive symptoms, particularly suicidal ideation, are challenging in the community context	‘We can ask all the questions, but they will kick us out if we ask, “do you have depression?” directly. They will say “who told you? get lost”…’ (Admin.) ‘The last question. about suicide, it will be difficult for them’ (Admin.)
**Intervention coherence** (the extent to which the participant understands the intervention and how it works)	Need to tailor intervention using education and multimedia, to enable better understanding	‘Sometimes when we talk to people directly, they won’t understand. Before giving questions to them we should give an overview of it. Tell them what is depression … once people are mentally prepared then we can ask them questions’ (Admin.) ‘Because some people have no understanding … it’s good to make them understand through the story, you get better response’
**Opportunity costs** (the extent to which benefits, profits or values must be given up to engage in the intervention)	Concern about family’s perception of opportunity costs	‘If we are using this app always, they [family members] will think that Self-help group people does not have any work to do that’s why they gave this, they are doing this instead of household chores’
**Perceived effectiveness** (the extent to which the intervention is perceived as likely to achieve its purpose)	Applications other than phone calls are perceived as useful Education and word of mouth can enhance perceived effectiveness	‘… because mobile has become part of life now, mobile is much needed because I have not purchased mobile just to keep it in pocket and roam now, for example if there is a program tomorrow I develop a notice in my mobile for that programme, I develop it and send directly to sir … we can do things like preparing notices, creating forms … I mean I need mobile for personal use and also for professional use. We pay for mobiles not just to make calls we should use it for everything’ (Admin.) ‘an awareness programme should be given to them. So even if [members who have used the app] when they say it to the people it is for their good, confidence is gained …’
**Self-efficacy** (the participant’s confidence that they can perform the behaviour(s) required to participate in the intervention)	Gender or age-based difference in self-efficacy	‘… people having less understanding or old people find it difficult’ ‘… in our organisation people who are older than 60 years – using smartphones is difficult for them – they use button phones’ (Admin.)
	Training and practice can increase self-efficacy	‘… we can give the tablet but before giving them tablet they should be trained properly, in training you can tell them how to use the tablet’ (Admin.) ‘Demo should be done after providing training’ ‘If we want to train them, they are already aged, with age, it becomes a little harder to use – initially when they gave it became little difficult, as we use it will become easy’
**Accessibility** (the participant’s ability to utilise the intervention without external constraints on when, where, how and how much they use it)	Accessibility of individual devices varies by age and gender	‘Everybody has but children and family member use sometime’ ‘We have mobile, it applies for everyone at home’ ‘Those who are in poverty they have mobile phones but still not available for girls’ (Admin.)
	Family support may be needed for improved accessibility	‘Whatever we do there [SHG] the family members may not encourage all those. When somebody else is doing this then they come and ask “why were the ladies given this?”’ (Admin.)

Quotes are from both administrators (Admin.) and self-help group (SHG) women.

#### Affective attitude

In general, we found a positive attitude toward mobile devices and the proposed intervention among participants, with some indication of needing to leave devices with a responsible individual for safekeeping. Participants were hesitant to use electronic devices at home due to lack of familiarity, and fear of damaging the device, preferring to defer the use of tablets to the group setting, with supervision from leadership:

‘There is a fear of ruining digital objects if they are touched.’

#### Burden

That is, the perceived amount of effort required to participate in the intervention, which was higher among older users or those with lower digital literacy:

‘… old people find it difficult’.

#### Ethicality

Overall, the intervention (app-based screening and brief behavioural intervention) was reported to fit well within the value system of SHG members. However, participants (administrators) noted that the question about suicidal ideation included in PHQ-9 was dissonant with the community setting and the purpose of their gathering. Participants reported on a previous project wherein high school students were asked PHQ-9 questions, and the teachers became offended to the extent that ‘they were about to hit us’:

‘The last question. about suicide, it will be difficult for them.’

#### Intervention coherence

Suggestions made to increase intervention coherence included providing education and using story-telling and multimedia to enhance understanding:

‘Tell them what is depression … once people are mentally prepared then we can ask them questions’.

#### Opportunity cost

Rather than their own concerns about opportunity costs, participants were concerned about their family members perceiving them to be wasting time if they were to use the app too frequently:

‘… they are doing this instead of household chores’.

#### Perceived effectiveness

Although SHG members’ expectations of perceived effectiveness may have been low at the outset, education and word of mouth can enhance perceived effectiveness:

‘When they say it to the people it is for their good, confidence is gained …’

#### Self-efficacy

Several participants noted that confidence in using the app varied with age, with older women having more difficulty participating in an app-based intervention. This could be addressed with both training and practice:

‘People who are older than 60 years – using smartphones is difficult for them.’

In addition to the above codes based on the theoretical framework of acceptability, the following new theme emerged:

#### Accessibility

We defined accessibility as participants’ ability to utilise the intervention without external constraints on when, where, how and how much they use it. We found that accessibility to mobile devices was variable across participants, based on age and gender. In general, women – specifically older women – appeared to have reduced access to smartphones and mobile devices. This theme emerged in both participant and administrator FGDs. Although the use of a collectivist device such as a tablet with a multiuser app could be a solution to this, FGD administrators indicated that women’s families may be a barrier to their access of the device or the intervention:

‘Those who are in poverty, they have mobile phones but still not available for girls.’

## Discussion

In India, as in other low- and middle-income countries, there is a huge gender gap in the ownership and patterns of mobile telephone and internet usage among women.^11^ The situation in rural areas is worse, where rural women’s access to mobile telephones is far lower than that of their urban counterparts.^12^ In rural India, despite the ‘freedom’ given to them (women can use telephones without needing permission), women were found to be subjected to significant constraints directly through (a) narrow expectations and desires around how women would use telephones; (b) women’s dependence on men for telephone ownership and lower proximity to phones; (c) the poorer functionality of women’s telephones; (d) women’s limited digital skills; and (e) time allocation constraints, wherein women had less leisure time and were subject to social norms that discouraged the use of a telephone for leisure.^13–15^ Similarly, we found that women in our study had low accessibility to mobile telephones, and the use a multiuser app in a community-based setting could address this barrier provided that family members were informed about the purpose of the app.

Mobile mental health is one way to address the large depression treatment gap in resource-constrained settings. However, available mental health apps often do not involve end-users in development and lack local language options and sociocultural relevance, and may not use audio and video formats to deliver content,^15^ limiting their usefulness. The MITHRA app is designed to address these limitations. It was developed in consultation with key end-users, is culturally appropriate (settings and characters in the video reflect the users), is available in the local language and is evidence based and client oriented.

Despite low accessibility, many of our participants demonstrated high self-efficacy in using digital interventions and a positive attitude towards mental health apps. This is perhaps a reflection of the initiative, Digital India, taken by the Indian government. This programme aims to make one person in every family e-literate, focusing on rural areas, by setting up state-wise training centres. An exploratory study on rural women in Vijayapura district (Karnataka) following implementation of this training found that many of the women perceived an improvement in their self-confidence (41.04%, *n* = 744) and knowledge (48.10%, *n* = 872) with regard to the use of mobile devices.^16^ However, we found age-related differences in self-efficacy with regard to digital interventions. Women older than 60 years of age were reported to use telephones only for making or receiving calls and were not familiar with smartphones. Training and technical assistance for these users could increase their confidence in using the app. Other features of the MITHRA app were developed specifically in response to feedback received in FGDs. For example, privacy was ensured by providing headphones, and accessibility was enhanced with audio versions of screening questions for illiterate users. Video modules were kept concise, respecting the limited free time available to the women, and were narrated in local languages to enhance engagement and comprehension.

There is limited evidence on the ethical issues involving the use of mental health intervention apps in rural India. Issues such as data privacy and confidentiality need to be explored, especially when deploying mental health apps in community-based settings. The inclusion of screening in mental health apps without paying attention to symptom severity may lead to stigma or unnecessary treatment-seeking behaviours, burdening already strained systems in low-resource settings.^17^ Given our participants’ opinion that questions about suicide feel incongruent in a community setting, in future iterations of the app we may use different depression screeners that do not include questions about suicidal ideation.

### Strengths and limitations

This qualitative study explored women’s knowledge of, and ease of using, smartphone applications, and gathered inputs on the design and features of the proposed app. These end-user inputs have helped us develop a mental health app that is responsive to the needs of the proposed audience for the app, culturally appropriate (settings and characters in the video), available in the local language, evidence based, client oriented and with content in both audio and video format. Although we conducted only three FGDs, we did observe data saturation and continued to obtain end-user input as a subset of FGD participants, as our PDG is engaged in further iterative modification of the app. One limitation is that women who consented to the FGD may also be inherently more likely to use app-based treatments. Additionally, FGDs were conducted by male researchers, and female participants may have hesitated to share their thoughts openly.

The MITHRA app is, to our knowledge, the first to deliver depression screening and brief psychological intervention for women in SHGs in India. MITHRA has been developed with end-user input and is a promising tool in the management of mild to moderate depression. We found that older women demonstrated lower self-efficacy in using mobile mental health apps, but all women had a positive attitude towards the app and depression interventions. With adequate training, and education of family members, a multiuser app installed on a tablet available in SHGs has the potential to identify and treat women with depression. Future research should examine the effectiveness of apps such as MITHRA in treating mild to moderate depression.

## Supporting information

Ruben et al. supplementary material Ruben et al. supplementary material

## Data Availability

The data that support the findings of this study are available on reasonable request from the corresponding author (A.B.). The data are not publicly available due to privacy/ethical restrictions.

## Supplementary material

The supplementary material for this article can be found at https://doi.org/10.1192/bjo.2025.8
