# Supplementary material for: Development of a Multiuser Interactive Health Response Application (MITHRA) for depression in women from a community-based organisation in India
Source: BJPsych Open. 2025 Mar 25;11(2):e58. doi: 10.1192/bjo.2025.8 (PMC12001939; doi:10.1192/bjo.2025.8)
Supplement: Ruben et al. supplementary material [file S2056472425000080sup001.docx]

**FGD discussion guide**

This focus group discussion guide is based on the theoretical framework of acceptability (Sekhon, M., Cartwright, M., & Francis, J. J. (2017). Acceptability of healthcare interventions: an overview of reviews and development of a theoretical framework. *BMC health services research*, *17*(1), 1-13.)


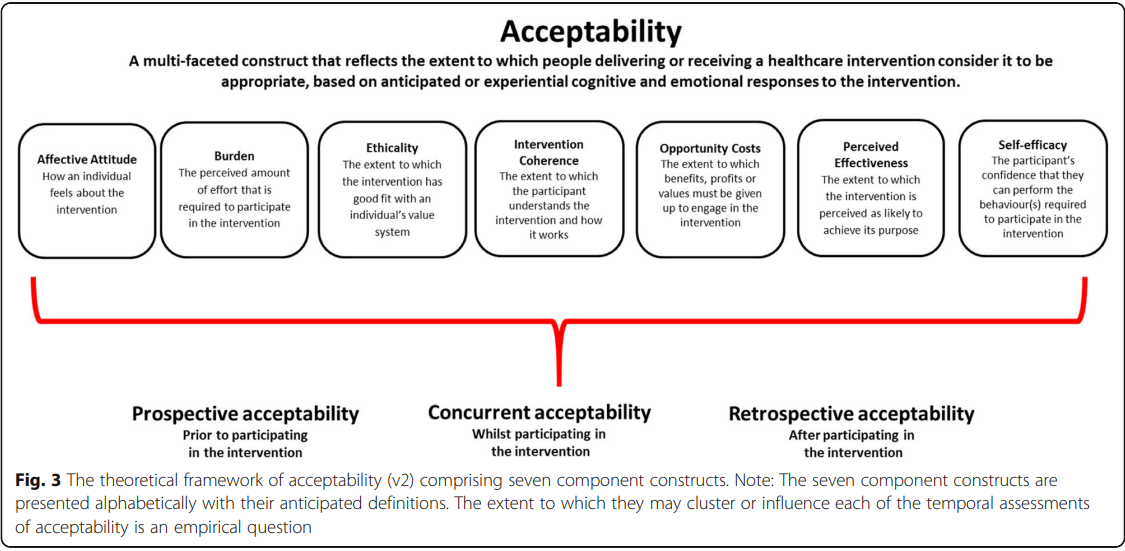


Instructions:

Collect demographic info including age

Introduction

1.    Informed   Consent

2.      Team and participant introduction

3.      Rapport building questions

a.      How are you?

b.      How are you feeling today?

c.      Have you had your food?

d.      Can I talk to you for some time?

4.      Basic General Physical Health

a.      How is your health?

b.      How do you rate your health (Rate card) - 0 to 5

                                                              i.          0-Bad

                                                             ii.          1-Very Poor

                                                            iii.          2-Poor

                                                            iv.          3-Good

                                                             v.          4-Very Good

                                                            vi.          5-Excellent

c.      What are the health problems you are having currently (if you are comfortable discussing this in a group)?

                                                              i.          List the problems

                                                             ii.          Are you taking treatment for these problems?

                                                            iii.          If yes, from whom?

                                                            iv.          Regular follow-up?

(Need a transition sentence here to the question about digital literacy.)

5.      Digital literacy

a.      Awareness of mobile and tablet devices

b. How common is ownership of personal smartphones? What do they use the smartphone for? (use of streaming videos etc to assess connectivity).

b.      Willingness to use

c.      Comfortable to use (with or without support)

6.      PHQ-9 Questions (Pictural and audio enabled)

a.      Would you be comfortable in using a depression screening questionnaire in pictorial and audio-enabled format in this tab/app? (Focus on Question 9 and get their input on it)

b. Would it be preferable for the PHQ-9 questions to be part of a general health screening questionnaire? (to reduce stigma)

7.      Based on your scoring, the app will give you options to use certain modules. These modules are basically developed to improve your depressive symptoms.

a.      How long would you like to view a module in a day?

                                                              i.          15 min

                                                             ii.          30 min

                                                            iii.          45 min

b.      Would you like to see the modules in the community or would you like to view it at your house

8. Do you think use of the app will reduce your ability to participate in other activities you usually do with this group? If yes can you give examples?

9. Do you think using the app can be helpful to reduce depression? Why or why not?

10. Would you share this information about the app with your family?
